# TITIN Regulates Body Size via the JH–JHE Pathway in Honeybees (*Apis mellifera*)

**DOI:** 10.3390/ijms27104501

**Published:** 2026-05-18

**Authors:** Xinying Qu, Hanbing Lu, Xinru Zhang, Lingjun Xin, Xiao Chen

**Affiliations:** 1 State Key Laboratory of Resource Insects, Institute of Apicultural Research, Chinese Academy of Agricultural Sciences, Beijing 100193, China; 82101235489@caas.cn (X.Q.); 821012530463@caas.cn (H.L.); 2 College of Bioscience and Resource Environment, Beijing University of Agriculture, Beijing 102206, China; 202330112016@bua.edu.cn (X.Z.); xinlingjun@bua.edu.cn (L.X.)

**Keywords:** JHE, JH, TITIN, RNAi, emergence weight, morphometric features

## Abstract

Honeybees play a vital role in pollinating crops and wild plants, but their health and efficiency are strongly influenced by their body size. Large bees tend to have longer lifespans, stronger foraging abilities, and greater resistance to diseases. However, the molecular mechanisms that control honeybees’ body size are not fully understood. In this study, we focused on 3 to 5-day-old larvae of *Apis mellifera ligustica* and investigated the roles and interactions of juvenile hormone (JH), juvenile hormone esterase (JHE), and TITIN in regulating honeybees’ body size using RNAi, exogenous hormone treatment, and qRT-PCR. The results showed that suppression of *Jhe* expression caused JH accumulation in larvae, subsequently reducing *Titin* expression and ultimately increasing adult body size. Furthermore, exogenous application of JHIII also inhibited the expression of *Titin*. Suppression of *Titin* expression alone directly increased the body size of adult honeybees, but did not affect the JH titer, which indicates that JH negatively regulates *Titin* expression in a unidirectional manner, whereas Titin does not feedback-regulate JH titer. The study suggests a regulatory link between JH, *Jhe*, and *Titin* in body size control. The discovery of this pathway, when combined with traditional breeding methods, may provide insights for future breeding strategies in honeybees.

## 1. Introduction

Honeybees, as highly social insects, are indispensable pollinators for global ecosystems and play an irreplaceable role in agricultural production [1,2,3]. Honeybees are holometabolous insects, meaning their individual development undergoes four distinct stages: egg, larva, pupa, and adult. The larval stage is particularly critical for caste differentiation. The conditions during larval development, especially nutrition, directly determine the physiological functions and morphological characteristics of the adult bees. Body size is a key trait affecting individual behavior and social division of labor in honeybees. Studies have shown that differences in workers’ body size (e.g., body weight, wing length) directly determine their foraging efficiency [4]. The enhanced carrying capacity of large workers for pollen and nectar thereby bolsters the colony’s collective food storage [5,6,7]. Body size is also closely related to physiological functions; for instance, large workers exhibit long lifespans and strong stress resistance [8,9].

Juvenile hormone (JH), a key regulator of insect metamorphosis and growth, has been demonstrated to affect honeybee development [10,11,12]. Studies have revealed that JH levels in queen and worker larvae exhibit two distinct peaks during the larval stage. The JH concentration at the first peak (around 4 days of age) is critical, as it determines whether the larva will develop into a queen or a worker [13]. The timing of the second JH peak (at 5 days of age) coincides with a critical window for ovarian morphological differentiation. During this stage, queen larvae undergo ovarian development through cell proliferation, whereas worker larvae experience programmed cell death in their ovaries, resulting in the loss of reproductive capacity [14,15]. Sustained high JH titers prolong the larval developmental phase, leading to increased body weight and size in the resulting adults [16]. These findings establish JH as a central hub integrating honeybee developmental programs with environmental signals.

The dynamic balance of JH is maintained through both synthesis (e.g., secretion by the corpora allata) and degradation pathways. A key enzyme in JH degradation is juvenile hormone esterase (JHE), which inactivates JH by catalyzing the hydrolysis of its ester bonds, thereby regulating JH titers in vivo [17,18,19]. Previous studies have demonstrated that JHE influences larval developmental duration and adult body size in insects such as *Drosophila* and silkworms by modulating JH levels [20,21]. However, the role of JH and JHE in structural implementation (muscle/cell growth) is less understood, and the link between endocrine signals and structural proteins is missing in honeybees.

In addition to hormonal pathways, body size development also relies on fundamental physiological processes such as cell growth and muscle development. TITIN, a giant structural protein widely present in animal muscle tissues, connects both ends of sarcomeres to maintain muscle fiber elasticity and length. Dysfunction of TITIN can directly impair muscle development [22]. In *Drosophila*, TITIN is encoded by the genes *Sls* and *Bt* (Projectin), both of which play critical roles in sarcomere development, stability, and the function of connecting filaments. Studies have shown that knockdown of *Sls* in the body wall muscles of *Drosophila* leads to altered muscle fiber morphology and severely disrupts neuromuscular junction formation [23], indicating that the functional state of *Titin* influences the establishment of muscle structures related to body size development in *Drosophila*.

Studies in brown planthoppers and wing-dimorphic crickets further indicate that elevated JH concentration can induce flight muscle degeneration [24,25]. As the largest and most critical structural protein in the sarcomere, *Titin* expression and stability are likely regulated during this process, thereby affecting muscle structure and function. However, in honeybees, the link between *Titin* and JH–JHE hormonal pathway remains unclear.

This study focuses on the critical developmental stage of honeybee larvae (3–5 days post-hatching). By employing RNAi technology to inhibit gene expression and qRT-PCR, and by measuring JH titer with an ELISA kit, it aims to determine the functional interactions among JHE, JH, and *Titin* in body size regulation. Furthermore, we seek to explore this novel regulatory pathway and decipher its underlying molecular mechanisms. These findings will contribute to a deeper understanding of the regulatory network governing honeybee growth and may identify potential targets for genetic improvement of body size in breeding programs.

## 2. Results

### 2.1. Inhibition of Jhe Expression Increased JH Titer and Suppressed Titin Expression

**Figure 1:**
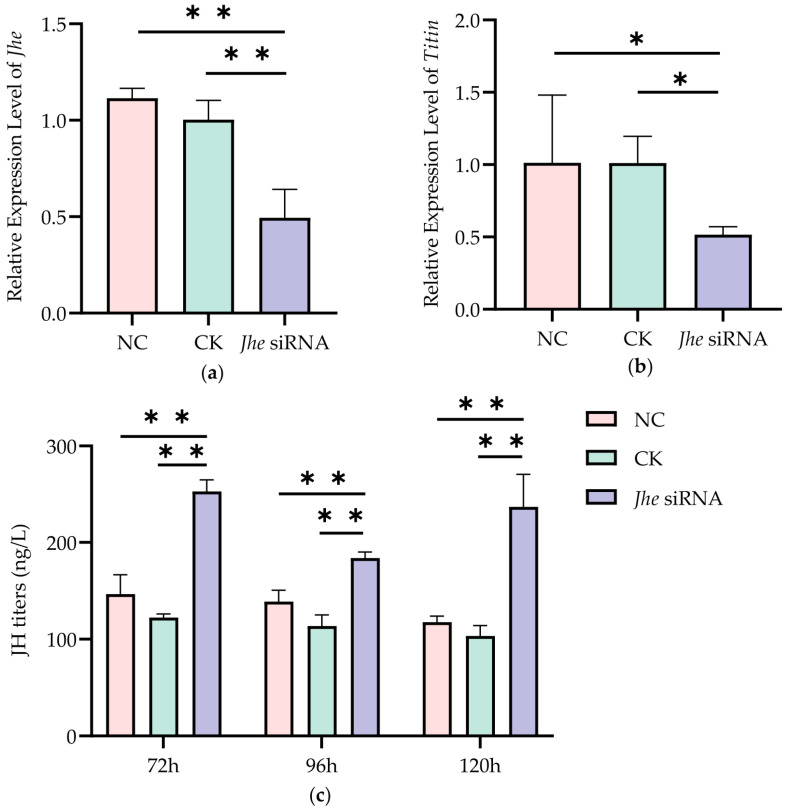
Changes in *Titin* expression level and JH titer following *Jhe* inhibition. (**a**) *Jhe* expression levels in worker larvae at 120 h. (**b**) *Titin* expression levels in worker larvae at 120 h. (**c**) JH titers in worker larvae at 72, 96 and 120 h. Note: * *p* < 0.05, ** *p* < 0.01, *n* = 5.

The experimental results showed that at 120 h of larval development, *Jhe* expression in the *Jhe* siRNA group was significantly lower than that in the CK group (*p* < 0.01) (Figure 1). Furthermore, *Titin* expression decreased by 48.42% ± 4.56% in the *Jhe* siRNA group compared to controls.

**Figure 2:**
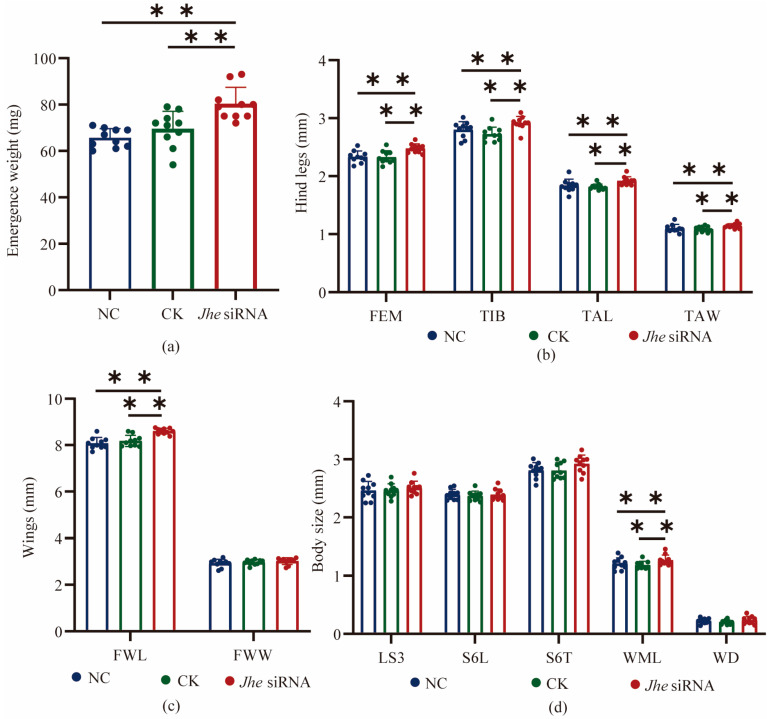
The changes of emergence weight and morphometric features after *Jhe* inhibition. (**a**) Emergence weight of workers. (**b**) Effects of *Jhe* knockdown on hind leg morphometric parameters. (**c**) Effects of *Jhe* knockdown on wing characteristics of workers. (**d**) Effects of *Jhe* knockdown on body size of workers. *Jhe* siRNA, *Jhe* siRNA groups; NC, negative control group; CK, blank control group. FWL, fore wing length; FEM, femur; TIB, tibia; TAL, basitarsus length; TAW, basitarsus width; WML, wax mirror of sternite 3 longitudinal; FWW, fore wing width; LS3, sternite 3 longitudinal; WD, distance between wax mirrors st. 3; S6L, sternite 6 longitudinal; S6T, sternite 6 transversal. Note: ** *p* < 0.01, *n* = 10.

We quantified JH titers in *Jhe*-suppressed larvae at three critical developmental stages (72 h, 96 h, and 120 h). The result showed JH levels in the *Jhe* siRNA group were significantly elevated (*p* < 0.01) compared with CK controls at all stages. Phenotypic characterization of adult bees demonstrated that *Jhe* suppression induced significant enlargement of multiple morphological features including FWL, FEM, TIB, TAL, TAW, and WML (Figure 2). Furthermore, we observed consistent increasing trends in FWW, LS3, WD, S6L, and S6T (not significant).

### 2.2. JHIII Suppresses Titin Expression

**Figure 3:**
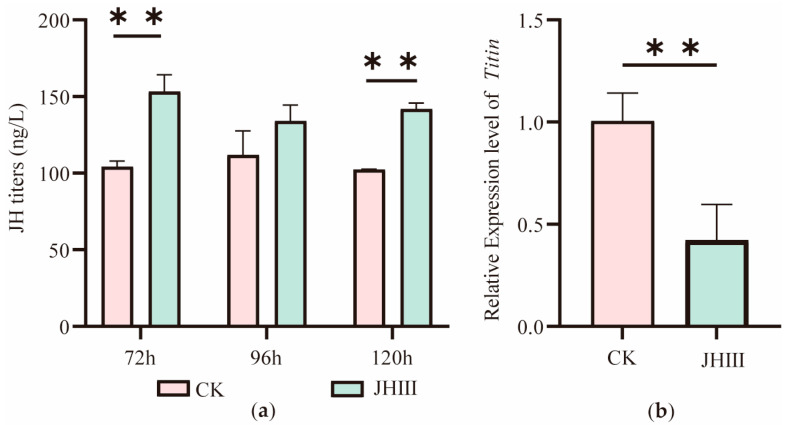
The changes of *Titin* expression and JH titer after JHIII supplementation. (**a**) JH titers in worker larvae at 72, 96 and 120 h. JHIII, In vitro JHIII supplementation group. CK, blank control group. (**b**) *Titin* expression levels in worker larvae at 120 h. Note: ** *p* < 0.01, *n* = 5.

We applied JHIII to the dorsal cuticle of honeybee larvae at 48 h of development. JHIII application increased JH titers at 72 h and 120 h (*p* < 0.05), while the increase at 96 h was not statistically significant (*p* = 0.058, Figure 3a). *Titin* expression was significantly reduced (57.82% ± 14.29%) in treated larvae compared to controls (Figure 3b). These results support a negative association between JH titers and *Titin* expression.

### 2.3. Suppression of Titin Affects Adult Honeybee Body Size

**Figure 4:**
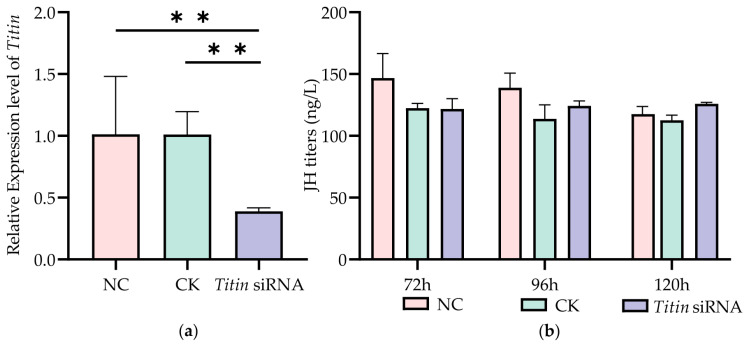
The changes of *Titin* expression and JH titers after *Titin* inhibition. (**a**) *Titin* expression levels in worker larvae at 120 h. (**b**) JH titers in worker larvae at 72 h, 96 h and 120 h. Note: ** *p* < 0.01, *n* = 5.

**Figure 5:**
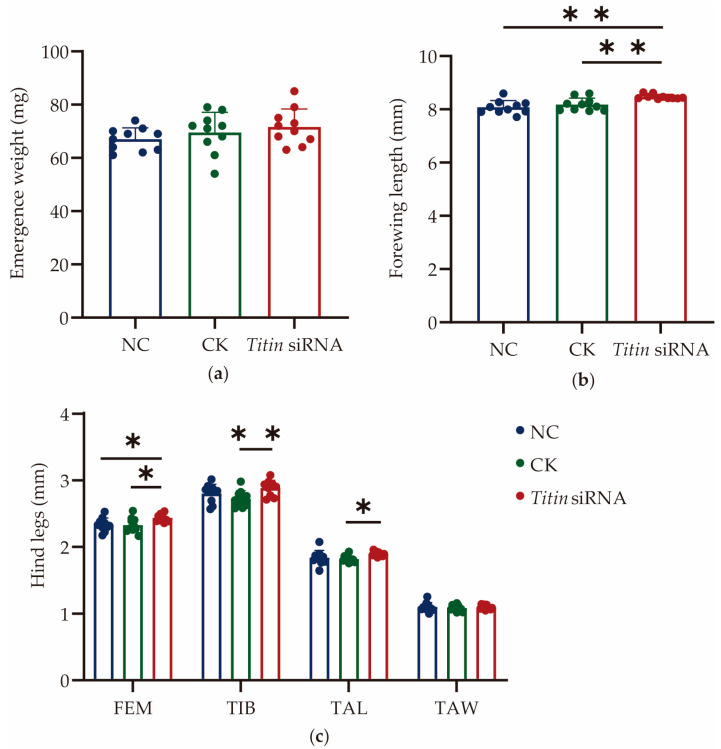

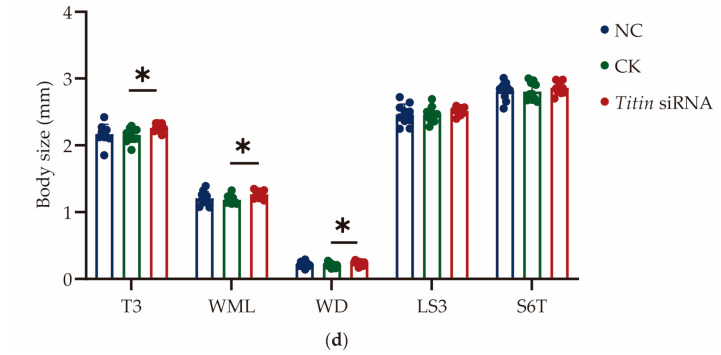
The changes of morphometric features after *Titin* inhibition. (**a**) Emergence weight of workers. (**b**) The changed morphological characteristics of forewing length of workers. (**c**) The changed morphological characteristics of hind legs of workers. (**d**) The changed morphological characteristics of body sizes of workers. *Titin* siRNA, *Titin* siRNA group. NC, negative control group; CK, blank control group. FWL, fore wing length; FEM, femur; TIB, tibia; TAL, basitarsus length; T3, tergite 3 longitudinal; WML, wax mirror of sternite 3 longitudinal; WD, distance between wax mirrors st. 3; TAW, basitarsus width; LS3, sternite 3 longitudinal; S6L, sternite 6 longitudinal. Note: * *p* < 0.05, ** *p* < 0.01, *n* = 10.

In this experiment, larvae were fed with the diet containing *Titin* siRNA at 48 h, 72 h, and 96 h, and measured the relative expression level of *Titin* in 120 h-old larvae (Figure 4). The results showed that compared with the CK group, *Titin* expression decreased by 61.05% ± 2.29% in siRNA-treated larvae (*p* < 0.01). However, the measurement result of JH titers at 72 h, 96 h, and 120 h showed no significant changes in the larvae treated with *Titin* siRNA. Morphological analysis showed significant increase in size in specific body parameters following *Titin* inhibition, including FWL, FEM, TIB, TAL, T3, WML, and WD (Figure 5). The emergence weight also exhibited an increasing trend, increasing by 3.02% compared with the CK group. These results indicate that *Titin* inhibition is associated with increased adult body size.

## 3. Discussion

In honeybees, body weight and body size are closely related to their health and production efficiency. It is worth noting that the emergence weight of workers has been confirmed to be closely related to their lifespan and physiological functions [26]. Larger workers generally exhibit longer lifespans, improved foraging abilities and enhanced stress resistance [8,9]. Therefore, enhancing the body weight and size of workers has become a key priority in honeybee breeding.

In this study, after *Jhe* inhibition, the JH titer at 72 h, 96 h, and 120 h of the larval stage significantly increased, and key morphological indicators of adult bees, such as FWL and FEM, significantly increased. This is consistent with the research conclusion of Negroni et al. [16], which states that a continuously high JH titer will prolong the larval development stage, thereby increasing larval weight and subsequent adult body size. In addition, the *Jhe* inhibition also leads to changes in adult bees’ body size. These results support a role for *Jhe* in regulating body size through modulation of JH titers. This echoes the reports in *Drosophila* and silkworms that *Jhe* affects adult body size by regulating JH titer [20,21], suggesting that the JH–JHE pathway plays important role in insects’ body size regulation.

Exogenous application of JHIII significantly elevated the JH titer in larvae (Figure 3b) and lead to a significant decrease in *Titin* expression (Figure 3a). Meanwhile, the JH accumulation caused by *Jhe* inhibition was also accompanied by a 48.42% decrease in *Titin* expression. This indicates that JH serves as an upstream negative regulatory factor of the *Titin* gene. Studies have shown that in locusts, JH can control the sex- and segment-specific maturation of muscle properties associated with reproductive behavior. Inactivating the corpora allata (CA) inhibits the maturation of these muscle properties, while injecting JH into the treated female locusts can reverse this effect. In honeybees, JH may also affect muscle-related proteins, including TITIN, through a similar mechanism [27]. As a key protein maintaining the structure and function of muscle fibers, reduced *Titin* expression may indirectly promote the increase of body size by affecting muscle development. In *Drosophila*, the knockdown of the *Sls* gene (TITIN homolog) leads to changes in muscle fiber morphology [23]. In this study, the body size of adult bees significantly increased after *Titin* inhibition (Figure 5), which further supports the role of *Titin* in body size construction. Although several linear measurements increased significantly following *Titin* inhibition, emergence weight showed only an increasing trend without statistical significance. This may suggest that *Titin* inhibition affects body proportions and morphometric allometry more strongly than total body mass. In addition, emergence weight measurements may exhibit greater biological variability than linear skeletal measurements, which could reduce statistical sensitivity.

The *Titin* inhibition experiment demonstrated that although the juvenile hormone (JH) titer during the larval stage remained unaffected (Figure 2b), adult morphological indicators such as the FWL and TIB still increased significantly (Figure 2c). This finding aligns closely with the core functional role of TITIN in muscle development—as a critical protein for maintaining the structural integrity of sarcomeres, the expression level of TITIN directly regulates the length and elasticity of muscle fibers [22]. Downregulation of its expression may delay the activation timing of adult muscle progenitor cells, leading to alterations in the organizational pattern of muscle tissue, thereby indirectly promoting the relative enlargement of adult bees’ body size [28]. It remains unclear how *Titin* down regulation mechanistically contributes to increased body size in honeybees. As a key structural protein of the sarcomere, TITIN may influence muscle architecture during metamorphosis by altering sarcomere length, modifying muscle attachment organization, or affecting myoblast proliferation and differentiation. These structural changes could ultimately contribute to enlarged adult body morphology. Future studies using immunofluorescence staining and transmission electron microscopy on honeybee flight muscles may help clarify the cellular and ultrastructural basis underlying this phenotype. Furthermore, our results demonstrated that JH negatively regulates *Titin* expression in a unidirectional manner. Elevated JH titers significantly suppressed *Titin* expression, whereas inhibition of *Titin* did not alter JH titers at any developmental stage examined. This indicates that JH acts upstream of *Titin* in the regulatory pathway, while *Titin* does not feedback-regulate JH titer, supporting the two form a unidirectional regulatory relationship of “JH → TITIN” in body size regulation, rather than a bidirectional interaction.

The growth and development of honeybee larvae is a highly complex process regulated by multiple factors [29,30]. This study demonstrated that *Jhe* inhibition during the larval stage leads to an increase in JH titer and a decrease in *Titin* expression, ultimately contributing to the formation of large adult honeybees. Suppression of *Titin* expression alone directly increased the body size of adult honeybees, but did not affect the JH titer, which indicates a unidirectional regulatory relationship between JH titer and *Titin* expression. These findings support a functional link between the JH–JHE axis and *Titin* in the regulation of body size. The results may provide a basis for future studies exploring breeding strategies. One limitation of the study is that RNAi silencing efficiency was evaluated only at 120 h, 24 h after the final siRNA administration. Because unmodified siRNA may exhibit limited stability in rapidly developing insects, the temporal dynamics of gene silencing at earlier intermediate stages (e.g., 96 h or 108 h) were not assessed. Future studies should incorporate additional sampling time points to better characterize the persistence and efficiency of RNAi effects during honeybee larval development.

## 4. Materials and Methods

### 4.1. Sampling

All samples were collected from *Apis mellifera ligustica* colonies. In May 2025, honeybee colonies were reared under standard beekeeping practices. To obtain age-synchronized larvae, the queen was confined to a specific comb using a queen cage for 12 h. On the fourth day after caging, larvae were selected from the brood comb and transferred to 48-well culture plates, with this time defined as 0 h. The larvae were subsequently reared in the laboratory according to the protocol established by Schmehl et al. [31], and a detailed composition and quantity of the artificial diet is provided in Table 1.

The larvae were divided into four groups: *Jhe* siRNA group (*n* = 35), *Titin* siRNA group (*n* = 30), the negative control group (NC group) (*n* = 65), and the blank control group (CK group) (*n* = 85) reared under normal conditions. At 48 h, 72 h, and 96 h, the *Jhe* siRNA group and *Titin* siRNA group were fed with diets containing 2 μg of their respective target-specific siRNAs, the NC group received a diet with 2 μg of nonsense sequence, and the CK group was fed a normal diet. The 2 μg siRNA dose was selected based on preliminary experiments showing effective gene knockdown without obvious larval mortality or developmental abnormalities. Samples for JH titer measurements were collected at 72 h, 96 h, and 120 h (always before feeding). Samples for gene expression analysis were collected at 120 h. The collected larval samples were flash-frozen in liquid nitrogen and stored at −80 °C for subsequent qRT-PCR and JH titer detection. At 144 h, the remaining larvae in the culture plates were transferred to a constant-temperature incubator (34 ± 1 °C, relative humidity 75% ± 5%, dark conditions) until adult emergence. Morphological characteristics of the emerged workers were then measured.

### 4.2. Inhibition of JHE and TITIN in Worker Larvae

To suppress the expression of *Jhe*, a siRNA sequence was synthesized (sense: 5′-GUU GCC CUA CAC GUA AUG UTT-3′; antisense: 5′-ACA UUA CGU GUA GGG CAA CTT-3′). To inhibit the expression of TITIN, a siRNA sequence was synthesized (sense: 5′-GGA UUG CAC GGU UGA GAA ATT-3′; antisense: 5′-UUU CUC AAC CGU GCA AUC CTT-3′), along with a nonsense control sequence (sense: 5′-UUC UCC GAA CGU GUC ACG UTT-3′; antisense: 5′-ACG UGA CAC GUU CGG AGA ATT-3′) (GenePharma, Shanghai, China). Feeding of siRNA and larvae sampling were implemented as described in the Sampling section.

### 4.3. RNA Isolation and Real Time Quantitative PCR

Total RNA was extracted from samples using the Trizol Up Plus RNA Kit (TransGen Biotech, Beijing, China, ER501-01-V2). Subsequently, 1 μg of RNA was reverse-transcribed into cDNA in a 20 μL reaction system using the PrimeScript™ RT reagent kit (TaKaRa, RR047A, Dalian, China). TITIN-specific primers (Table 2) were designed using Oligo 6.0 software and synthesized by Sangon Biotech (Shanghai, China) Co., Ltd. The expression levels of *Jhe* and *Titin* in worker larvae were then quantified by qRT-PCR using the TB Green Premix Ex Taq II kit (TaKaRa, RR820A) and analyzed using the LineGene 9600 Plus Real-Time PCR System (Bioer Technology, Hangzhou, China).

The cDNA samples were serially diluted (1, 10^−1^, 10^−2^, 10^−3^, 10^−4^) to determine the optimal dilution factor, which was ultimately established as 2 × 10^−1^. The PCR protocol consisted of initial denaturation at 95 °C for 30 s, followed by 45 cycles of 95 °C for 5 s (denaturation) and 60 °C for 30 s (annealing/extension), with simultaneous fluorescence signal acquisition for amplification and melting curve generation. This process not only determined the optimal sample dilution ratio but also verified primer specificity.

For gene expression detection, reactions were performed in a 20 μL total volume containing 1 μL cDNA template (200 ng, 1:5 dilution), 0.8 μL forward primer (10 μM), 0.8 μL reverse primer (10 μM), 10 μL premix, and 7.4 μL H_2_O. *β-actin* served as the reference gene, with cycling conditions identical to preliminary experiments. The 2^−ΔΔCt^ method was employed for systematic analysis of Ct values obtained from qRT-PCR experiments.

### 4.4. JH Treatment and Assay

Worker larvae were reared in the laboratory following the methods described in Section 2.1. At 48 h of larval development, hormone treatment was implemented. We dissolved 10 μg of JHIII (Macklin) in 1 μL methanol for each individual larval treatment a dose previously demonstrated to effectively elevate JH titers in worker bees [33]. The control group consisted of normally reared larvae (CK group). For the experimental group, the JHIII-methanol solution was topically applied to the dorsal cuticle of 48 h-old worker larvae. The larvae were then allowed to develop until collected. Samples were collected at three stages (72 h, 96 h, and 120 h) for JH titer quantification, with distinct sample processing protocols at each stage: at 72 h, three larvae were pooled as one biological sample; at 96 h, two larvae constituted one sample; while individual larvae were processed separately at 120 h. Three biological replicates were set up for each stage, and each sample was subjected to three technical replicate detections using ELISA (Multiskan go, Thermo Scientific, Waltham, MA, USA). Since JH III is the predominant juvenile hormone homolog in honeybees, the detected ELISA signal was considered to mainly reflect JH III levels. During sample preparation, each larva was rinsed with sterile water to remove any unabsorbed JHIII from the body surface. Subsequently, PBS buffer was added at a ratio of 1:9 (*w*/*v*), followed by vortex mixing and centrifugation (3000 rpm, 20 min). Aliquots of the resulting supernatant (10 μL each) were collected and analyzed using a commercial ELISA kit (Shanghai Renjie Biotechnology, Shanghai, China) to determine the JH titer. The ELISA kit used in the study had a reported minimum detection limit of <0.1 ng/L according to the manufacturer’s specifications. All measured JH titers in this study were above this detection threshold. In addition, similar ELISA-based methods have previously been applied for juvenile hormone quantification in honeybees, supporting the suitability of this approach for honeybee samples [34].

### 4.5. Morphological Measurement of Workers

**Figure 6:**
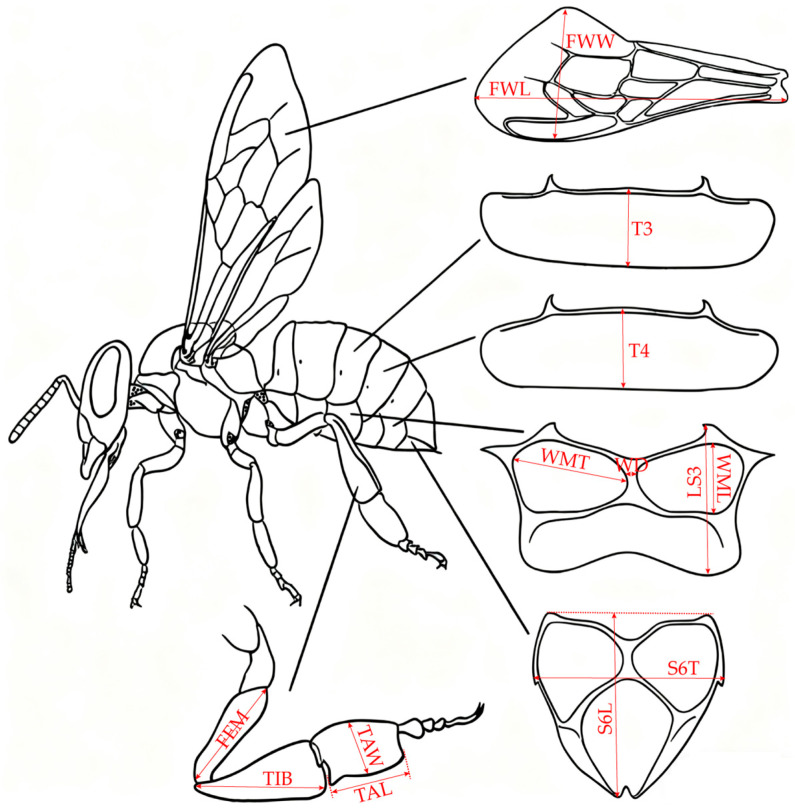
Schematic diagram of honeybee morphological index measurement. FWL, fore wing length; FWW, fore wing width; T3, tergite 3 longitudinal; T4, tergite 4 longitudinal; WMT, wax mirror of sternite 3 transversal; WD, distance between wax mirrors st.3; LS3, sternite 3 longitudinal; WML, wax mirror of sternite 3 longitudinal; S6L, sternite 6 longitudinal; S6T, sternite 6 transversal; FEM, femur; TIB, tibia; TAW, basitarsus width; TAL, basitarsus length.

Worker bees were sampled right after their eclosion, and their eclosion emergence weight was quantified with a precision electronic balance. Afterwards, referring to the experimental protocols established by Ruttner and Meixner [35,36], workers individuals were selected for the determination of morphological traits. The measured parameters included fore wing length (FWL), fore wing width (FWW), femur (FEM), tibia (TIB), basitarsus length (TAL), basitarsus width (TAW), tergite 3 longitudinal (T3), tergite 4 longitudinal (T4), sternite 3 longitudinal (LS3), wax mirror of sternite 3 longitudinal (WML), wax mirror of sternite 3 transversal (WMT), distance between wax mirrors st. 3 (WD), sternite 6 longitudinal (S6L), and sternite 6 transversal (S6T) (Figure 6). All morphological images were captured using a LEICA DMS300 digital microscope at a uniform magnification. After image acquisition, Adobe Photoshop 2023 software was used to determine the actual sizes of the morphological characteristics of worker bees.

### 4.6. Data Analysis

In the analysis of qRT-PCR results, β-actin was used as the reference gene to correct differences between samples. The relative expression level of the target gene was calculated using the 2^−ΔΔCt^ method. Specifically, the difference in Ct values between the target gene and the reference gene (ΔCt = Ct value of the target gene − Ct value of β-actin) was first calculated for each sample. Then, using the ΔCt value of the CK group as the reference baseline, the ΔΔCt value was calculated. Finally, the relative expression level of the target gene was determined using the formula 2^−ΔΔCt^, which reflects the trend of gene expression changes.

In the analysis of JH titer results, first, a standard curve was plotted with the standard concentration as the abscissa (*x*-axis) and the corresponding OD value as the ordinate (*y*-axis). The concentration of each sample was calculated according to the curve equation. Then, the mean value and standard deviation of JH titers in workers of each group were calculated. For the analysis of morphological traits, the mean values and standard deviations of 14 morphometric parameters (FWL, FWW, FEM, TIB, TAL, TAW, T3, T4, LS3, WML, WMT, WD, S6L, S6T) were computed for each worker group. All statistical analyses were performed using GraphPad Prism 10.1.2 software. Data are presented as mean ± SD. Statistical significance was defined at α = 0.05. For two-group comparisons (CK vs. treatment, as in Figure 1, Figure 3 and Figure 4): a two-tailed Student’s *t*-test for independent samples was used. For three-or-more-group comparisons (CK vs. NC vs. siRNA, as in Figure 2 and Figure 5): a one-way ANOVA followed by Tukey’s HSD post-hoc test or pairwise comparisons was used.

## 5. Conclusions

This study focused on 3 to 5-day-old larvae of *Apis mellifera ligustica* and systematically investigated the roles and relationships of JH, *Jhe*, and *Titin* in regulating honeybee body size. The results showed that inhibition of JHE significantly increased the JH titer in larvae, inhibited *Titin* expression, and ultimately led to an increase in the body size of adult honeybees. The exogenous application of JHIII also down regulated *Titin* expression. In contrast, though suppression of *Titin* directly increases the body size of adult honeybees, it did not affect the JH titer. This supports the hypothesis about the unidirectional relationship in which JH negatively regulates *Titin* expression, whereas *Titin* does not feedback-regulate JH titer. These findings support a functional link between the JH–JHE axis and *Titin* in the regulation of body size. The results may provide a basis for future studies exploring breeding strategies.

## Figures and Tables

**Table 1:** Amount and percentage of diet components in the larval diet.

Larval Age (*n*th Day)	Feed Composition					Feed Amount
	Royal Jelly (%)	Glucose (%)	Fructose (%)	Yeast Extract (%)	Sterile Water (%)	
1	44.25	5.3	5.3	0.9	44.25	20 μL/larvae
2	/	/	/	/	/	0
3	42.95	6.4	6.4	1.3	42.95	20 μL/larvae
4	50	9	9	2	30	30 μL/larvae
5	50	9	9	2	30	40 μL/larvae
6	50	9	9	2	30	50 μL/larvae

**Table 2:** Primer sequences for qRT-PCR of *Jhe* and *Titin*.

Primer Name	Primer Sequence	Ref.
*Jhe*-F	5′-CTTTTCTCGCTTCCACAACC-3′	Designed by this study [32]
*Jhe*-R	5′-TCCTGGTCCAGCAATGTGTA-3′	
*Titin*-F	5′-ACGTACGAGTGCGATGACG-3′	
*Titin*-R	5′-CTCCGTCGTTTCGACCAGTT-3′	
*β-actin*-F	5′-CTGCTGCATCATCCTCAAGC-3′	
*β-actin*-R	5′-GAAAAGAGCCTCGGGACAAC-3′	

## Data Availability

The original contributions presented in this study are included in the article material. Further inquiries can be directed to the corresponding author.

## Abbreviations

The following abbreviations are used in this manuscript:

FEM	Femur
FWW	Fore wing width
JH	Juvenile hormone
JHE	Juvenile hormone esterase
LS3	Sternite 3 longitudinal
S6L	Sternite 6 longitudinal
S6T	Sternite 6 transversal
T3	Tergite 3 longitudinal
T4	Tergite 4 longitudinal
TAL	Basitarsus length
TAW	Basitarsus width
TIB	Tibia
WD	Distance between wax mirrors st.3
WML	Wax mirror of sternite 3 longitudinal
